# Can relief measures nudge compliance in a public health crisis? Evidence from a kinked fiscal policy rule^☆^

**DOI:** 10.1016/j.jebo.2022.08.020

**Published:** 2022-10

**Authors:** Claudio Deiana, Andrea Geraci, Gianluca Mazzarella, Fabio Sabatini

**Affiliations:** a University of Cagliari and CRENoS, Italy, and University of Essex, UK; b European Commission, Joint Research Centre (JRC) Ispra, Italy; c University of Pavia, Italy; d Sapienza University of Rome, Italy, and IZA, Bonn, Germany

**Keywords:** Compliance, Civic capital, COVID-19 policy response, Stay-at-home orders, Regression kink design

## Abstract

We show that compensation measures aimed at improving the fairness of a crisis policy response can unintendedly nudge compliance with emergency rules. We combine information on the distribution of relief funds across Italian municipalities during the novel coronavirus pandemic with data tracking citizens’ movements through mobile devices and navigation systems. To assess the impact of transfers on compliance, we exploit a sharp kink schedule in the allocation of funds. The empirical analysis provides evidence that compliance increased with transfers, suggesting that the observance of emergency rules also depends on the fairness of the pandemic policy response.

## 1 Introduction

Extraordinary threats to public welfare require an effective state capable of enacting emergency measures promptly. The exercise of coercive power is only one of the factors of state effectiveness. A broader view gives reciprocal obligations between institutions and citizens an essential role (Besley, 2020). Enlightenment thinkers such as Locke (1690) and Rousseau (1762) first argued that people accept obligations in return for benevolent government. This view relates the willingness to comply with rules to the belief that authorities and social arrangements are appropriate and fair, as if a “psychological contract” with institutions was in force (Tyler, 1990, Feld, Frey, 2007). The strength of the contract varies across citizens, with more civic-minded individuals having a stronger propensity for reciprocity that makes them more reactive to fairness (Besley, 2020).

The novel coronavirus outbreak offers a convenient scenario for studying the relationship between fairness and voluntary compliance. In a pandemic, the spontaneous observance of emergency rules is crucial to public welfare. At the onset of the outbreak, citizens were required to comply with sudden limitations of their civil liberties that were not compensated with any evident public good equivalent in the short run. Confinement measures can be perceived as unbearably unfair to anyone needing social interaction to get an income, especially those who can neither work from home nor afford food delivery. Economically vulnerable individuals face the most challenging difficulties in coping with lockdown rules and have more substantial incentives to break social distancing norms (Wright, Sonin, Driscoll, Wilson, 2020, Jung, Manley, Shrestha, 2021). A pandemic policy response that asks the poor to stay at home without helping them satisfy their essential needs is likely to be perceived as unsustainable and unfair, undermining compliance with the emergency rules.

In this paper, we show that measures mitigating the economic disruption caused by social distancing could unintendedly nudge compliance with restrictive orders. To address causality, we exploit the sharp kink design in the allocation of funds of a relief program explicitly aimed at improving the fairness of the pandemic policy response. On March 30, 2020, the Italian government announced an aid scheme to support economically vulnerable groups through the distribution of food stamps. We combine information on the allocation of resources across Italian municipalities with data tracking citizens’ movements through mobile devices and navigation systems, drawn from City Analytics by Enel X. As social distancing requires renouncing unnecessary movements, we follow the literature and use human mobility as a proxy for compliance (e.g., Allcott, Boxell, Conway, Gentzkow, Thaler, Yang, 2020, Bargain, Aminjonov, 2020, Barrios, Benmelech, Hochberg, Sapienza, Zingales, 2021, Durante, Guiso, Gulino, 2021, Egorov, Enikolopov, Makarin, Petrova, 2020, Chen, Frey, Presidente, 2021, Painter, Qiu, 2021). For each municipality, we obtain information about mobility differentials relative to pre-crisis levels (between January 13 and February 16, 2020). The granularity of the data allows us to measure the observance of restrictions at the highest possible level of disaggregation for Italy.

The kink design in the allocation of funds as a function of past income differentials generated a random treatment assignment in a neighborhood of the threshold point, which allows us to tackle endogeneity issues. Authorities partitioned the budget of the program, 400 million euros (≃485 million dollars), into two quotas. Eighty percent was distributed to municipalities proportionally to their population. The remaining amount was allocated as a function of the difference between the municipal and the national per capita income in 2017, weighted by the population share.

We first illustrate the sharp kink schedule in the distribution of funds. In a neighborhood of the threshold point, access to funds relied on the random deviation of the municipality per capita income from the national level in 2017. Standard tests support the validity of the assumptions by showing no manipulation of the assignment variable at the kink. In the neighborhood of the threshold point, all covariates are balanced, and there are no changes in slope or spurious kinks due to nonlinearities.

We then assess the impact of the relief program on compliance through a Regression Kink Design (RKD). We find robust evidence that, after the introduction of the program, the deviation of mobility from its baseline level increased substantially with the amount of transfers. In the week of the policy announcement, the transfers caused an increase in the mobility differential from the baseline of 3 percentage points. Given an average drop of 60 percentage points in the same week, additional 0.58€ additional transfers per capita caused a 5 per cent increase in compliance. Two weeks later, the impact of transfers on compliance was still positive, statistically significant, and sizable, with the increase in transfers leading to an increase in mobility differentials of roughly 3.5 percentage points.

Standard and more recently developed RKD robustness checks show that our results resist irrespective of the bandwidth choice, with stronger effects holding in the proximity of the cut-off and the coefficient stabilizing with distance from the threshold. We show that municipal features that may affect mobility are smooth in a neighborhood of the threshold point. A placebo analysis in the spirit of the permutation test of Ganong and Jäger (2018) supports the causal interpretation of results.

The heterogeneity of the effects suggests that the impact of transfers on compliance is driven by the improvement in the fairness of the pandemic policy response. As civic-minded citizens have a stronger propensity for reciprocity in their relationship with the state (Besley, 2020), we test whether more “civic” areas manifested a stronger reaction to transfers. We show that in municipalities with a lower civic capital, the effect of the additional transfers is not statistically significant. In municipalities where civic culture is stronger, the relief program caused a significant increase in compliance, with treatment effects following the same pattern observed in our main specification.

Our work relates to several strands of literature. First, we connect to studies explaining that compliance results from reciprocal obligations tying citizens to institutions (Tyler, 1990, Smith, 1992, Murphy, Tyler, 2008). We provide empirical evidence suggesting that the relationship between policy fairness and compliance also holds in a specific, but of general interest, scenario, the novel coronavirus pandemic. The extant literature mostly approaches the “contractarian view” (Besley, 2020) from a theoretical perspective (Tyler, 2006, Feld, Frey, 2007, Besley, 2020), or by empirically assessing the drivers of a specific type of compliance, related to tax obligations, through field experiments (e.g., Hallsworth, List, Metcalfe, Vlaev, 2017, Kogler, Olsen, Bogaers, 2020, De Neve, Imbert, Spinnewijn, Tsankova, Luts, 2021, Mascagni, Mengistu, Woldeyes, 2021). We add to this field by testing the impact of a policy action aimed at improving the fairness of the crisis management on the observance of emergency rules. Our results overall support the contractarian view, suggesting that people’s willingness to follow the rules also depends on the fairness of institutions.

We also contribute to the growing body of research analyzing the drivers of social distancing such as civic capital (Barrios, Benmelech, Hochberg, Sapienza, Zingales, 2021, Durante, Guiso, Gulino, 2021), social norms (Casoria et al., 2021), partisanship (Allcott, Boxell, Conway, Gentzkow, Thaler, Yang, 2020, Simonov, Sacher, Dubé, Biswas, 2021, Painter, Qiu, 2021), public role models (Abel, Brown, 2020, Ajzenman, Cavalcanti, Da Mata, 2020), ethnic diversity (Egorov et al., 2020), economic preferences (Müller and Rau, 2020), social trust (Bargain and Aminjonov, 2020b), expectations (Briscese et al., 2020), and information (Mendolia et al., 2021). Our results add to this field by suggesting that fiscal policies may also play a role, and authorities could leverage targeted relief programs to incidentally nudge compliance.

Finally, we relate to the COVID economics literature by analyzing the outcomes of policies intended to mitigate the pandemic economic disruption. Previous research addresses the impact of fiscal support for payroll and fixed costs (Alstadsæter et al., 2020) and tracks consumers’ response to COVID-19 fiscal stimuli (Bayer, Born, Luetticke, Müller, 2020, Coibion, Gorodnichenko, Weber, 2020, Kaplan, Moll, Violante, 2020, Kubota, Onishi, Toyama, 2021). Green and Loualiche (2021) exploit nonlinearity in the award of federal grants to US state governments to estimate the state and local employment response to federal stimulus. In a paper close in spirit to ours, Fetzer (2020) shows how a program stabilizing the public’s demand for dining out contributed spreading the viral disease. Areas that benefited from the program most also registered a remarkable increase in the emergence of new infection clusters, due to lesser social distancing. Our evidence adds to this literature by directly addressing the stimulus’ impact on social distancing. Our results complement Fetzer’s finding that stimulating the demand for the hospitality sector can worsen the epidemiological situation. Targeting relief programs at economically disadvantaged groups may potentially exert an opposite effect through an increase in compliance with social distancing recommendations.

The remainder of the paper is organized as follows. Section 2 provides context and describes our data. Section 3 shows the kink schedule in the Italian government’s relief program and illustrates our empirical strategy. In Section 4, we present and discuss our results. Section 5 concludes.

## 2 Context and data

In this section, we first provide some background (Section 2.1). We then illustrate the sharp kink schedule in the food relief program (2.2). Finally, we describe our mobility data (2.3).

### 2.1 Background

**Fig. 1:**
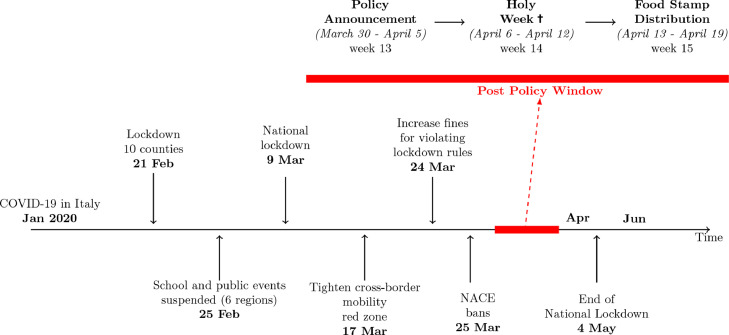
Timeline of the outbreak in Italy.

On March 9, 2020, Italy was the first Western democracy to impose a national lockdown, requiring the confinement of the population at home. Authorities closed schools, restaurants, and non-essential shops and banned any outdoor activity, including walking far from home. Citizens could only leave their houses for a handful of reasons— for example, to go to the supermarket or the pharmacy—and needed to carry a document stating the reason they were outside.^1^ Fig. 1 illustrates the timeline of the outbreak in Italy.

Though helpful in flattening the contagion curve (Amuedo-Dorantes, Borra, Rivera Garrido, Sevilla, 2020, Flaxman, Mishra, Gandy, Unwin, Mellan, Coupland, Whittaker, Zhu, Berah, Eaton, Monod, Team, Ghani, Donnelly, Riley, Vollmer, Ferguson, Okell, Bhatt, 2020), confinement measures were *de facto* the most substantial suppression of constitutional rights in the history of the Republic, undermined the incumbent government’s popularity (Fazio et al., 2022), and threatened the social contract between citizens and the state. Lockdowns triggered economic hardship for millions of people, with unskilled workers and the poor bearing the heaviest toll (Wright et al., 2020). Mongey et al. (2020) show that workers with less ability to work from home suffered higher unemployment increases and were less able to comply with stay-at-home orders. Palomino et al. (2020) find sizable changes in poverty and inequality across Europe, with Southern countries witnessing the highest rise and increases varying with the duration and intensity of social distancing measures. The authors estimate an average increase in the headcount poverty index from 4.9 to 9.4 percentage points and a mean loss rate between 10% and 16.2% for the working poor.

Colussi (2020) documents that the industrial production fell by almost 30% in Italy and GDP contracted by 4.7% in March 2020 due to lockdown measures. In April, the industrial production further contracted by 19.1% relative to March. The welfare system and the suspension of layoffs delayed the impact of the crisis on the labor market. The unemployment rate fell by 11,1% in March 2020 and continued decreasing in April due to the massive increase in economically inactive people. Requests for subsidies for temporary reductions of hours worked (*Cassa Integrazione Guadagni*) increased by about 2,953% with respect to April 2019. According to the Italian National Institute for Social Security (*Istituto Nazionale della Previdenza Sociale*, INPS), 51% of Italian firms, accounting for 40% of the private sector employment, adopted short-time work schemes in March and April 2020. Their employees reduced the hours worked by about 90% and experienced a 27% loss in their gross monthly wage (Bovini et al., 2020). Figari and Fiorio (2020) show that 41% of those who suffered from earning losses belonged to one-earner households: for them, the temporary shutdown of their activities caused the loss of the primary income source.

To soften the economic consequences of restrictive measures, the government initially launched a 25-billion-euros package (*Cura Italia* Decree on March 17) with the aim of preserving employment and income levels. The Decree temporarily banned layoffs, extended unemployment benefits for employees in every productive sector independently of business size, introduced a lump sum transfer targeted at self-employed and seasonal workers, and provided wage compensation for workers facing an involuntary reduction in working hours (the beneficiaries of the Wages Guarantee Fund, *Cassa Integrazione Guadagni*). In late May, after lifting most of the restrictive measures, the government passed a new 155 billion-euros stimulus package (Relaunch Decree on May 15), which reinforced and extended many of the *Cura Italia* measures, and introduced new transfers schemes to support families and businesses.

Brunori et al. (2021) show that the stimulus packages largely neutralized the short-term impact of restrictive measures on the poorest households. Nonetheless, the crisis and the government’s policy response did not affect all households equally, with workers at the bottom of the wage distribution and those less able to work from home facing the most challenging difficulties, especially after emergency measures were lifted (Aina, Brunetti, Mussida, Scicchitano, 2021, Bonacini, Gallo, Scicchitano, 2021, Carta, De Philippis, 2021).

Despite one of the stimulus packages, the *Cura Italia* Decree, was launched shortly before the aid scheme we address in our work - The Food stamps program - its impact cannot confound the results of the empirical analysis. First, the Food stamps program targeted a different population, also including inactive workers. Second, it followed a unique, kinked, allocation rule that had no equal in the *Cura Italia* Decree transfers. In the next section (2.2), we provide information on the Food stamps program. In Section 2.3, we describe the allocation mechanism in detail, showing the sharp kink design that regulated the distribution of funds.

### 2.2 The food stamps program

Social distancing orders resulted in a dramatic increase in the demand for food and financial aid from the most disadvantaged groups. Towards the end of March 2020, tension mounted in more impoverished areas, with police patrolling supermarkets following a series of thefts. To cope with the mounting discontent, the government announced new relief measures lending € 4.3 billion (≃5.2$ billion) to the municipal solidarity fund and allocating 400 additional millions (≃485 million dollars) to an emergency relief program providing food stamps to economically disadvantaged groups.^2^ The government stressed the intention to levy these measures to make the COVID-19 policy response fairer, create a buffer against the increase in poverty, and halt social unrest. In our empirical analysis, we focus on the 400 million food aid program as the authorities allocated part of the funds following a kink design (Section 2.3) that allows us to identify the effect of transfers.^3^ The Civil Protection Department partitioned the program’s budget into two quotas. Eighty percent (*Quota* A) was distributed to municipalities proportionally to their population. The remaining amount (*Quota* B) was allocated as a function of the difference between the municipality and the national per capita income in 2017, weighted by the population share.^4^

The Civil Protection Decree 85/2020 (March 30, 2020) provided the general guidelines for the distribution of funds across municipalities.^5^ Local administrations could use the assigned resources to provide food stamps and shopping vouchers, retaining the discretionary power to establish their values and define the targeted population.^6^

For example, the municipality of Rome delivered the financial support to disadvantaged households that could not afford their basic needs and were not entitled to other kinds of transfer. Households with (strictly) less than three members were entitled to food stamps for an overall value of 300€, those between three and four members received food stamps for 400€, and families larger than five received 500€.^7^

The municipality of Milan adopted similar criteria and delivered a 150€ voucher to households with up to three members and a 350€ voucher to families with more than three members. Potential beneficiaries were then ranked based on the criteria we show in Table A.2 in the Appendix. In case of identical scores, authorities gave priority to families with i) children 0–3 years old, ii) members aged over 75, iii) lower family income; and iv) lower value of the first house.

**Table 1:** The food stamps allocation criteria around the threshold.

Panel A: Boltiere			Panel B: Levate	
Above the threshold			Below the threshold	
Household members	Imports		Household members	Imports
1	150		1	150
2	250		2	250
3	300		3	325
4	350		4	375
5	400		5	425
6	450		6	475
7	450		6	475
8	450		6	475

*Note:* The table shows the allocation criteria in a municipality above (Boltiere) and below (Levate) the thresold we exploit in our identification strategy (Section 2.3). We draw the data from the public calls for applications.

The municipality of Naples allocated food stamps weekly. Each voucher amounted to 100€, for a total amount of 300€ over three weeks. Beneficiaries had to spend the 100€ voucher in a single shot to avoid repeated visits to supermarkets and limit social contacts.

In principle, municipalities above the threshold, receiving higher resources, could allocate the extra funds to increase the scheme coverage or the voucher value, or both. However, the available evidence suggests that the municipalities in the neighborhood of the cut-off adopted similar allocation criteria. To give an example, we show in Table 1 the criteria adopted in two municipalities in the Province of Bergamo (Lombardy), one of the most affected areas during the first wave of the outbreak. The table displays the criteria for the distribution of food stamps adopted in Boltiere, a municipality lying above the threshold, and Levate, which was below the threshold.

The criteria were similar, with a one-member household receiving food stamps for 150€, a two-member household receiving 250€, and a three-member family receiving 300€ in the municipality above the threshold and 325€ in the one below the threshold. Overall, the aid scheme succeeded in giving financial support to the families in need. Municipalities only slightly differentiated the value of vouchers and the population coverage. Eligibility was subject to income testing, and food stamps requests were subject to random ex post controls. Anecdotal evidence suggests that all the assigned funds were effectively distributed by local administrations and spent by beneficiaries. After its termination, the program was refunded twice in December 2020 and February 2021.^8^

### 2.3 The kinked policy rule

In this section, we describe the kink schedule in the distribution of funds. To reconstruct the allocation mechanism, we combine information about the amount received by each municipality under the two quotas, provided by the Civil Protection Department, with data on 2017 income taxes aggregated at the municipality level, provided by the Department of Finance.^9^

We compute the municipal per capita income, $I_{m}$, as total taxable income of the municipality in 2017 divided by the municipal population. The national income, $I_{N}$, is the sum of taxable incomes in all municipalities in 2017 divided by the Italian resident population. According to the Decree of the Head of the Civil Protection Department, each municipality m should receive the following amount under the two quotas, *A* and *B*:

(1) $$Q_{A,m}=\frac{pop_{m}}{pop_{N}}Q_{A,tot}$$

(2) $$Q_{B,m}=\left\{ \begin{array}{cc} \psi \;\frac{pop_{m}}{pop_{N}}Q_{B,tot}\;\left(D_{m}-c\right) & \text{if}\;D_{m}\geq c\\ 0 & \text{if}\;D_{m}<c \end{array}\right.$$

where, $pop_{m}$ is the resident population in municipality $m,pop_{N}$ is the total population resident in Italian municipalities. $D_{m}=\left(I_{N}-I_{m}\right)$ is the difference between national and municipal per capita income. $Q_{A,tot}$ and $Q_{B,tot}$ is the total budget of the aid program.^10^ Parameter c is the cut-off value for the income gap, while ψ is a rescaling factor ensuring that $\sum_{m}Q_{B,m}=Q_{B,tot}$:

(3) $$\psi ={\left(\sum_{m}\left(\left(D_{m}-c\right)\frac{pop_{m}}{pop_{N}}1_{\left\{D_{m}>c\right\}}\right)\right)}^{-1}$$

Putting the two quotas together, the per capita amount of transfers received by municipality m can be summarized as:

(4) $$q_{m}=\left\{ \begin{array}{cc} \underset{\alpha_{0}}{\underset{︸}{\frac{Q_{A,tot}}{pop_{N}}}}+\underset{\alpha_{1}}{\underset{︸}{\psi \;\frac{Q_{B,tot}}{pop_{N}}}}\;\left(D_{m}-c\right) & \text{if}\;D_{m}\geq c\\ \frac{Q_{A,tot}}{pop_{N}} & \text{if}\;D_{m}<c \end{array}\right.$$

The above formula describes the kinked policy schedule. Below the cut-off, c, the per capita amount of transfers is fixed at $\alpha_{0}=Q_{A,tot}/pop_{N}$ and identical across municipalities. Above the cut-off, the amount increases linearly with the gap between the national and the municipal income, $\alpha_{1}=\psi \;\left(Q_{B,tot}/pop_{N}\right)$.

We compute the parameters of the kink design $\left\{c,\alpha_{0},\alpha_{1}\right\}$ combining information on transfers and municipality income taxes. The value of the cut-off c above which municipalities are entitled to receive the extra funding via quota B is neither clearly indicated in the Decree nor in any related official document. We retrieve this value from the data as the minimum amount of the gap between the municipality and the national per capita income $I_{m}$ observed in the subsample of municipalities entitled to quota B, equal to -1466€. This value means that quota B transfers benefited the municipalities whose deviation from the average per capita income did not exceed 1466€, with the amount of transfers increasing with the distance from the cut-off.

Having defined the parameter c, we apply Eq. (3) to compute the value of ψ, and then obtain the parameter $\alpha_{1}$, which is equal to 0.586, using Eq. (4). This value measures the per capita amount of the quota A transfers received by each municipality, 5.302€.

**Fig. 2:**
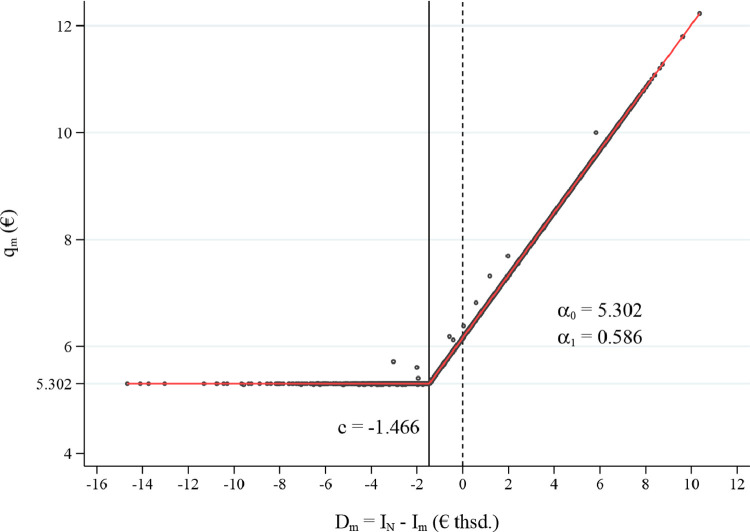
The kinked allocation mechanism. *Note:* The graph shows the empirical relationship between the amount of transfers per capita, and the difference between municipal and national per capita income (in € thsd.). Since $D_{m}=I_{N}-I_{m}$, municipal average income decreases moving from the left to the right. Municipalities on the right of $D_{m}=0$ are municipalities with an average income below the national average. The slope of the kink schedule is computed using Eq. (3).

In Fig. 2, we plot the empirical relationship between the per capita amount of transfers received by each municipality, $q_{m}$, and the computed difference between the national and the municipal per capita income, $\left(I_{N}-I_{m}\right)$, expressed in € -thousands. The figure also shows, in red, the kink schedule and its parameters. The per capita income of municipalities on the left of the origin is higher than the national average, i.e., the deviation of the municipality from the average is negative.

**Fig. A.1:**
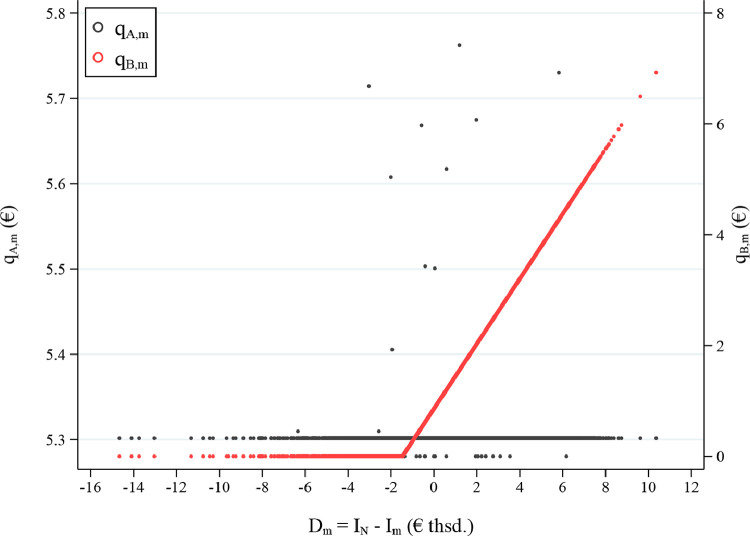
The relationship between $q_{A,m},\;q_{B,m}$ and $\left(I_{N}-I_{m}\right)$. *Note:* The graph shows the empirical relationship between the per capita amount of transfers, separately for the two components A and B, and the difference between the municipal and national per capita income (in € thsd.). Each dot represents a municipality, separately for $q_{A,m}$ (in black) and $q_{B,m}$ (in red). (For interpretation of the references to colour in this figure legend, the reader is referred to the web version of this article.)

As expected, the kink schedule identified by Eq. (4) is sharp. The per capita amount of transfers is approximately equal to 5.3€ ($\alpha_{0}$) for all municipalities with an income gap smaller than -1466€ (c).^11^ Above the cut-off, transfers increase linearly with the the gap between the national and municipal per capita income, with a computed slope equal to 0.586 ($\alpha_{1}$). Figure A.1, in the Appendix, shows the relationship between each of the two components - A and B - and the computed difference between the national and the municipal per capita income.

Importantly, since the national-municipal income gap is expressed in € -thousands, the slope coefficient ($\alpha_{1}$) implies that a one unit increase in the difference between the national and the municipal income – equal to 1000€ – generates additional per capita transfers for 0.586€. These additional transfers correspond to an 11% increase of the resources to be distributed to beneficiaries, with respect to municipalities below the cut-off ($\alpha_{1}/\alpha_{0}=0.586/5.302≃0.11$).

**Fig. 3:**
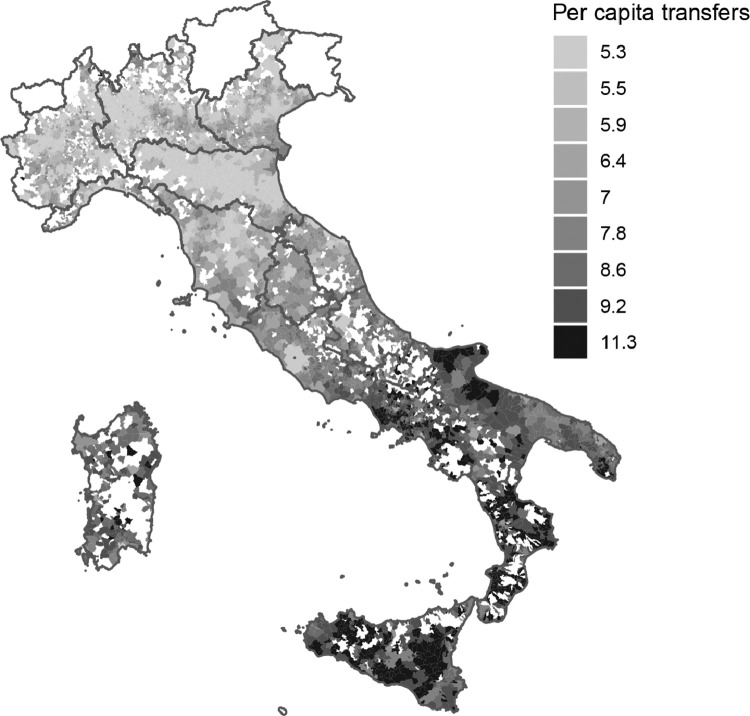
Distribution of the total amount of transfer across municipalities.

Figure 3 illustrates the distribution of total transfers per capita (i.e. accounting for both “Quota A” and “Quota B”) across Italian municipalities. Shades of gray illustrate the heterogeneity in the distribution of funds, with darker areas receiving higher amounts. White areas cover the three autonomous regions that benefited from different aid schemes and are therefore excluded from the analysis. Since funds are allocated based on past municipal incomes weighted by the population, transfers are higher in the South of Italy than the North.

### 2.4 Mobility

As social distancing requires renouncing to unnecessary movements and staying at home as much as possible, the literature has used indicators of mobility to measure compliance at the city (Ajzenman, Cavalcanti, Da Mata, 2020, Egorov, Enikolopov, Makarin, Petrova, 2020), province (Durante et al., 2021), region (Bargain and Aminjonov, 2020b), or US county level (Allcott, Boxell, Conway, Gentzkow, Thaler, Yang, 2020, Barrios, Benmelech, Hochberg, Sapienza, Zingales, 2021, Simonov, Sacher, Dubé, Biswas, 2021). Previous studies on Italian mobility during the lockdown employed province-level indicators drawn from Teralytics (Durante et al., 2021), Cuebiq Inc. (Pepe et al., 2020), or Google Mobility Reports (Caselli et al., 2020).

To measure compliance, we follow the literature and exploit data from Enel X to build an indicator of mobility at the municipality level. Enel X data rely on the geolocation system of mobile and vehicles navigation devices to capture individual movements and aggregate them at the municipal-daily level.

Mobility is expressed as the percent deviation of the ratio between citizens’ movements and the municipal population from the baseline level observed before the pandemic crisis from January 13, to February 16, 2020.^12^

**Fig. 4:**
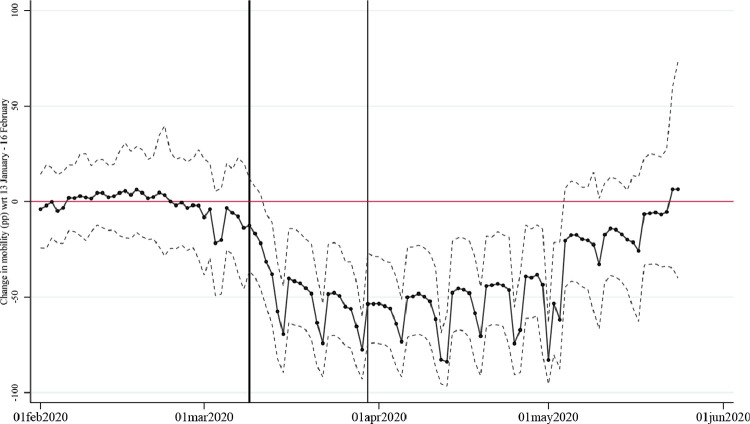
Average daily change in mobility in Italy. *Note:* The graph shows the pattern of the mobility index over the period February 1-June 1. The thicker vertical line represents the beginning of the lockdown while the thin solid line indicates the policy announcement. Source: our elaboration on Enel X data.

Figure 4 illustrates the change in mobility between February 1, and May 31, 2020. Each dot represents the average daily change in mobility across Italian municipalities. The two dotted lines represent the daily mobility trends observed in the 10th and the 90th percentile of the national distribution of mobility.

The plot shows that mobility rapidly decreased with the beginning of the lockdown on March 9, (thick solid line). A further decline occurred after March 21, when the government tightened confinement measures by shutting down all non-necessary businesses and industries. The lockdown formally ended on May 4, but pre-crisis levels were reached only towards the end of May. The series also follows clear weekly patterns, with working days showing higher mobility and downward peaks representing the substantially smaller movements on the weekends and Easter holidays.

## 3 Empirical strategy

In this section, we first describe our regression kink design for assessing the impact of the aid program on mobility across the Italian municipalities (3.1). Then, we describe our estimation sample and the covariates we control for in the empirical analysis (3.2).

### 3.1 The kink design

As described in the previous section, the per capita amount of transfers above the cut-off point is a deterministic linear function of the difference between the national and the municipal income.

We exploit the exogenous change in slope at the cut-off point (kink) to identify the causal effect of transfers on compliance with stay-at-home orders, as measured by the mobility index described in Section 2.4.

Given the linear dependence of the per capita amount of transfers and municipal income above the cut-off point, an OLS estimate of the relationship between mobility and transfers would likely be biased, because municipal income may be correlated with unobservable determinants of mobility.

To solve this potential endogeneity issue, we adopt the standard *Regression Kink Design* (RKD) firstly introduced by Nielsen et al. (2010), and more recently developed by Card et al. (2015) and Simonsen et al. (2016). The key intuition is that if the amount of transfers affects compliance, then the relationship between the outcome and the forcing variable will also show a change in slope at the cut-off point. The identifying assumption is that in a neighborhood of the cut-off point, the change in the amount of transfers induced by the kink is as good as random.

Define as τ the ratio of the discontinuity in derivatives at the cut-off point in the outcomes Y divided by the discontinuity in the amount of benefits received by each municipality:

(5) $$\tau =\frac{{\lim_{d\to c+}\frac{\partial E[Y|D=d]}{\partial d}|}_{d=c}-{\lim_{d\to c-}\frac{\partial E[Y|D=d]}{\partial d}|}_{d=c}}{\lim_{d\to c+}Q^{\prime }\left(d\right)-\lim_{d\to c-}Q^{\prime }\left(d\right)},$$

where Q(.) is a function that maps the amount of benefits received and the value of the running variable D. As showed by Card et al. (2015), the quantity described in (5) identifies the “treatment-on-the-treated” parameter introduced by Florens et al. (2008) or, equivalently, the “local average response” parameter introduced by Altonji and Matzkin (2005) at the threshold point c, i.e. the extent to which the outcome Y will vary in response to a unit variation in the amount of the received treatment.

In our case, the quantity at the denominator of Eq. (5) comes from the deterministic function Q(.) and we do not need to estimate it, i.e., we are in a sharp RKD. This quantity is equal to 0.586, i.e. the $\alpha_{1}$ parameter in Fig. 2. On the other hand, the numerator can be identified by the coefficient $\beta_{1}$ of the following equation:

(6) $$Y_{wm}=\beta_{0}+\beta_{1}\left[Z_{m}\times \left(D_{m}-c\right)\right]+\beta_{2}f\left(D_{m}-c\right)+\varepsilon_{wm},$$

where $Y_{wm}$ is the mobility index of municipality m in week w. $D_{m}$, the difference between national and municipal income per capita is the running variable and c is the cut-off. $Z_{m}=1_{\left\{D_{m}\geq c\right\}}$ and $f\left(D_{m}-c\right)$ includes a polynomial function of the running variable and their interactions with $Z_{m}$.

We estimate Eq. (6) using the optimal bandwidth proposed by Calonico et al. (2019a) (CCT hereafter) separately for each week. We use a triangular kernel and a linear polynomial. Since nonlinearity in the relationship between the outcome and the forcing variable might generate a spurious kink at the the cut-off point we show that similar results are obtained using a second order polynomial. In our preferred specifications, we also include municipality-level controls to capture residual variability, allowing the bandwidth to change accordingly (see Calonico et al., 2019b).

It is important to stress that, by assumption, our RKD setup identifies the treatment effect of the transfers on mobility only in a neighborhood of the cut-off. Differently from a standard Regression Discontinuity Design (RDD) setting, the treatment in the RKD is inherently continuous. This implies that above the the cut-off, it is not the probability of being treated that changes. Instead, what changes is the intensity of the treatment. This difference has implications for the interpretation of the coefficient of interest, $\beta_{1}$, in Eq. (6).

The $\beta_{1}$ coefficient measures the change in mobility – $Y_{wm}$ – for every unit increase (1000€) in the difference between national and municipal income – $D_{m}$ in a neighborhood of the cut-off. Since the slope of the allocation mechanism above the cut-off ($\alpha_{1}$) implies a 0.586€ additional per capita transfer for a one unit increase in the running variable, under the RKD assumption we can also interpret $\beta_{1}$ as the effect of 0.586€ additional per capita transfers on mobility in a neighborhood of the cut-off.

**Fig. 5:**
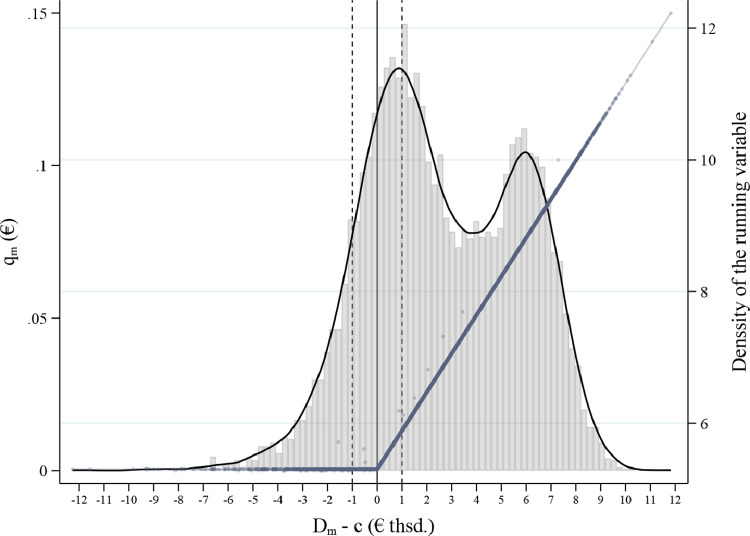
Kink schedule and the distribution of the running variable. *Note:* The figure shows the distribution of the re-centered running variable $D_{m}-c=\left(I_{N}-I_{m}-c\right)$ (left y-axis) against the kink schedule for $q_{m}$ (right y-axis). The running variable is re-centered such that the cut-off is equal to 0 (black solid line). The vertical dashed lines define the interval [−1;1] around the cut-off.

In Fig. 5 we plot the distribution of the re-centered running variable $\left(D_{m}-c\right)$ – right y-axis – against the kink schedule for $q_{m}$ – left y-axis. Notice that in this graph, due to the re-centering of the running variable, the cut-off is equal to 0. Roughly 23% of the sample, corresponding to approximately 1650 municipalities, are in the interval [−1;1].

### 3.2 The estimation sample

We start with 7257 municipalities in the 17 (out of 20) Italian regions for which it was possible to match the data on the aid program transfers with municipality tax records.^13^ As mentioned in Section 2.3, we use aggregated municipal tax records to compute the forcing variable we employ to estimate the kink.

We aggregate daily mobility data for each municipality at the weekly level. We keep only municipality-week observations for which the daily mobility index is non-missing for the entire week. We also drop provinces where all municipalities are either all above or all below the cut-off of the kink design. After putting together the municipality-week panel, the final sample consists of 47997 municipality-week observations for 5333 municipalities observed between the 10th week (March 9–15) and the 18th week 18 (May 4–18).

Finally, we complement our dataset with a set of municipality-level covariates that could affect mobility but have no relationship with the kink, as confirmed by the results of the balancing test.

We use ISTAT 2011 Census data to control for municipalities’ population and degree of urbanization, as people living in small towns may have to travel higher distances to reach the workplace or satisfy basic needs.

Since the essential activities that remained open during the lockdown are a source of mobility, we also control for the number of active firms and the number of workers in 3-digits NACE categories using data from the 2018 ISTAT register of Active Enterprises (ASIA - Enterprises). Specifically, we compute an indicator of firm density as the number of firms divided by the municipality’s surface. We also build an indicator of the share of essential workers over the population. We define the essential NACE categories following the classification proposed by Di Porto et al. (2020) for Italy.

Recent economic studies suggest that compliance with confinement measures also is a matter of social capital (Bargain, Aminjonov, Barrios, Benmelech, Hochberg, Sapienza, Zingales, 2021, Borgonovi, Andrieu, 2020, Durante, Guiso, Gulino, 2021). Healthy people may not properly understand the risks of contagion, and infected individuals with mild symptoms do not derive any personal benefit from staying at home. The willingness to contribute to community’s welfare and the belief that most people will do the same may be a relevant incentive to comply (Barrios et al., 2021). As pointed out by Barrios et al. (2021), this combination of “values and beliefs that help a group overcome the free-rider problem” is what Guiso et al. (2010) define as civic capital and the literature commonly labels as social capital (Putnam et al., 1993). We use the ISTAT 2011 census of nonprofit institutions to construct a municipality-level indicator of social capital that captures the per capita number of Putnam-type associations, including civil and human rights, civil protection, environmental, international cooperation and solidarity, philanthropic, social cohesion, and social service organizations.^14^

Finally, we control for the change in mobility during the first week of the lockdown, which provides the baseline level of compliance observed in each municipality.

### 3.3 Testing the validity of the design

As suggested by Card et al. (2015), there are two testable necessary and sufficient conditions for the validity of the RKD. The first is the smoothness condition of the density of the forcing variable. Evidence of bunching of observations around the cut-off point could signal endogenous sorting and cast doubts about the validity of the design. In our context, the forcing variable is a function of the distribution of municipal incomes in 2017, more than two years before the beginning of the pandemic. Therefore, it is implausible to observe endogenous sorting around the threshold.

**Fig. 6:**
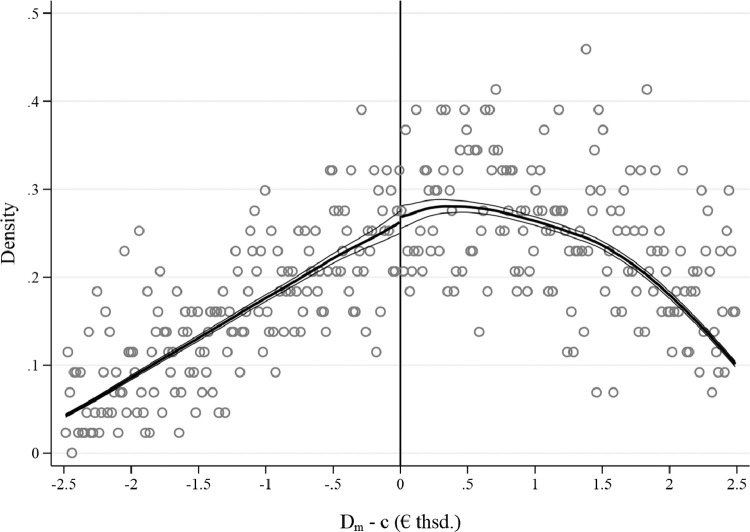
Smoothness of the conditional density of the running variable. *Note:* The figure shows the density of the running variable in a neighborhood of the cut-off point using the graphical test proposed by McCrary (2008).

Figure 6 provides evidence of smoothness in the conditional distribution of the running variable around the cut-off point based on the graphical test proposed by McCrary (2008). In the figure the running variable is centered on the cut-off, $\left(D_{m}-c\right)$ on the the x-axis.

The second testable condition requires the conditional distribution of the pre-determined covariates to be smooth around the cut-off point. To test for the smoothness of the first derivative of covariates’ conditional expectation functions, we estimate separate kink regressions for each municipality-level covariate we use in our preferred specifications, using both a linear and a quadratic polynomial. The results in Table 2 show no changes in slope neither with a linear (column 1) nor with a quadratic polynomial (column 2). Importantly, results show the absence of a kink for the change in mobility during the first week of lockdown (the 10th week of 2020), providing an implicit placebo test since the policy was introduced at the beginning of the 13th week.

**Table 2:** Smoothness of pre-determined covariates.

	Polynomial order	
	Linear	Quadratic
Covariate	(1)	(2)
Population	3023.966	1853.324
	(3938.087)	(8618.253)
Municipality area	-0.5161	-0.5649
	(6.5784)	(12.0341)
Urban area dummy	-0.0278	-0.0165
	(0.0638)	(0.1303)
Firm density	8.7910	5.3742
	(5.6603)	(14.4248)
Share of essential workers per capita	-0.0075	-0.0096
	(0.0105)	(0.0208)
Density of non-profit organizations	0.0583	0.1438
	(0.0648)	(0.1391)
Change in mobility during the first week of lockdown	-0.0150	-0.0236
	(0.0154)	(0.0258)

*Note:* The table shows tests for the presence of changes in the slope of the conditional expectation of covariates determined before the treatment assignment. The test is performed using both a linear and quadratic polynomial (columns 1 and 2 respectively). For each kink regression the bandwidth is computed according to CCT. Robust standard errors in parentheses. *p<.10, **p<.05, ***p<.01.

## 4 Results

We first present our results about the impact of the relief program on compliance across Italian municipalities (4.1). We then provide a battery of robustness and placebo tests to support the causal interpretation of results (4.2). Finally, we assess the heterogeneity of the effects providing evidence that suggests that the fairness channel is empirically relevant (4.3).

### 4.1 Main results

**Fig. 7:**
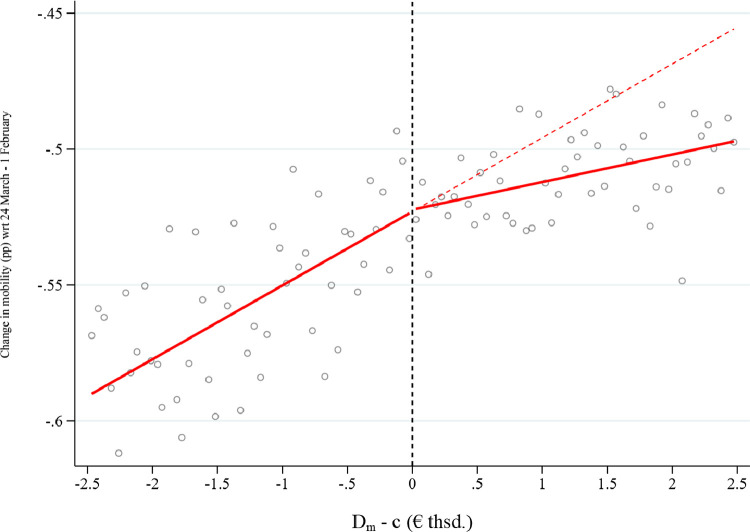
RKD plot - pooling pre and post weeks. *Note:* The figure shows the cross-sectional relationship between mobility and the running variable. The plot is obtained pooling observations from the week of the policy announcement (week 13 of 2020) to the end of stay-at-home orders (May 4, 2020, i.e., week 18). The running variable is binned using 100 symmetric bins. The segmented red line is obtained estimating separately two linear regressions using observations below and above the cut-off point. The dashed red line above the cut-off corresponds to the extrapolation of the regression line below the cut-off. (For interpretation of the references to colour in this figure legend, the reader is referred to the web version of this article.)

We start by providing a graphical representation of the cross-sectional relationship between mobility and the running variable of the kink design, i.e., the gap between the national and the municipal per capita income. While, as described in the previous section, the kink parameter β from Eq. (6) will be estimated separately for each week, the plot in Fig. 7 is obtained pooling observations from the week of the policy announcement (week 13 of 2020) to the end of stay-at-home orders (May 4, 2020, i.e., week 18). We also subset observations within a reasonably small bandwidth (2.5) to characterize the empirical relationship in a neighborhood of the cut-off point, and bin the running variable using 100 symmetric bins.

The first thing to notice in Fig. 7 is the existence of a positive relationship between mobility and the running variable. This implies that poorer municipalities are, on average, less compliant with confinement measures. This evidence is consistent with previous findings that poorer regions comply less with shelter-in-place policies, as low-income agents need not interrupt income-generating activities to escape poverty and hunger (Bargain, Aminjonov, Dasgupta, Jonsson Funk, Lazard, White, Marshall, 2020).

More importantly, a change in slope is visually evident at the cut-off. The negative change in slope suggests that the treatment negatively affects mobility across municipalities.

**Fig. 8:**
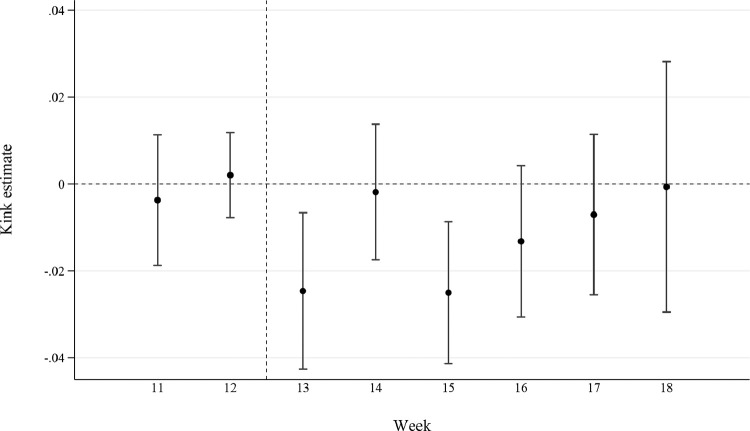
Program’s impact on mobility - pre and post policy announcement. *Note:* The figure shows the kink estimates and the associated standard errors using the CCT optimal bandwidth and a linear polynomial. Results are displayed for the weeks preceding the policy announcement (11 and 12) and the weeks after (13 to 18). Week 11 refers to March 16–22, week 12 to March 23–29, week 13 to March 30-April 5, week 14 to April 6–12, week 15 to April 13–19, week 16 to April 20–26, week 17 to April 27-May 3, and week 18 to May 4–10.

We now turn to the estimates of the treatment effects. Figure 8 shows the results of our preferred specification, with a linear polynomial, CCT optimal bandwidth, and including a set of predetermined municipality-level covariates. The figure shows the treatment effects from the 11th week of 2020 (i.e., two weeks before the program announcement) to the 18th week.

The two lockdown weeks before the program announcement (week 11 and week 12) serve as a placebo test in the spirit of Card et al. (2015). The coefficients are virtually zero in magnitude and never statistically significant, suggesting that, before the announcement, there was no relationship between mobility and the amount of funds that municipalities received two weeks later.

According to our main specification (column 2 of Table 3 and column 2 of Table A.1), in the first week following the program announcement (March 30 - April 5), we observe that 0.58€ per capita additional transfers cause an increase in compliance, i.e., in the mobility differential respect to the baseline level, of 2.5 percentage points.^15^ Given an average drop in mobility of roughly 60 percentage points in the same week, the 2.5 percentage points reduction induced by the extra transfers corresponds, approximately, to a further 4% reduction in mobility.

**Table 3:** Kink estimates - main.

			Mobility Index		
	Linear models			Quadratic models	
	(1)	(2)		(3)	(4)
$\beta_{1}^{13}$	-0.0344**	-0.0246**		-0.0466*	-0.0332**
	(0.0170)	(0.0110)		(0.0258)	(0.0144)
CCT Bandwidth	1.2616	1.3787		2.4413	3.0649
N	1721	1792		2751	3048
Covariate	No	Yes		No	Yes
$\beta_{1}^{15}$	-0.0400**	-0.0250**		-0.0490*	-0.0353*
	(0.0159)	(0.0099)		(0.0259)	(0.0182)
CCT Bandwidth	1.3043	1.5968		2.3791	2.6659
N	1752	2009		2708	2849
Covariate	No	Yes		No	Yes

*Note:* The table contains coefficients and standard errors showing the estimated effect in percentage points increase of weekly mobility at the vicinity of the threshold point. The coefficients $\beta_{13}$ and $\beta_{15}$ refer to 13th (March 30, - April 5) and the 15th week (April 13–19), respectively. The bandwidths are selected using the procedure by Calonico et al. (2019a). Robust standard errors in parentheses. *p<.10, **p<.05, ***p<.01.

In the 14th week the treatment effect shrinks in magnitude and becomes not statistically distinguishable from the reduction in mobility caused by the closure of essential activities and the tightening of lockdown enforcement for the Holy Week.

In the third week after the program announcement, when authorities actually distributed most food stamps, and the aid scheme found the highest resonance in the media, the additional transfers cause a decrease in mobility similar to the one estimated for week 13, equal to approximately 2.5 percentage points. The impact of the program then gradually stabilizes over the subsequent weeks, becoming indistinguishable from the reduction in mobility observed in the municipalities just-below the cut-off.

The fading of the effect from the end of April is expected for several reasons. First, authorities ended the distribution of food stamps. The new Economic and Financial Document (*Documento di programmazione economica e finanziaria*) approved on April 24 replaced the transfers scheme addressed in our empirical analysis with new relief measures following different allocation rules. As the cut-off ceased to play a role in the allocation of funds, we cannot observe the treatment effects in its neighborhood anymore. In addition, the government lifted most restrictions from May 4 (see Fig. 1), allowing mobility for work and health reasons and visits to relatives within and across municipalities in the same region. Sport centers, parks and cemeteries re-opened, and manufacturing, construction, real estate brokerage and wholesale activities gradually resumed with the requirement to respect social distancing. As a result, the variation in mobility became indistinguishable above and below the cut-off.

The duration of the effect is consistent with previous findings that cognitive and affective nudges are generally short-lived, differently from nudges that imply a persistent change in individual behavior. For example, environmental nudges can persist over time to the extent to which they encourage investments in technology adoption, such as the purchase of energy-efficient durable goods (e.g., Allcott and Rogers, 2014). Instead, cognitive and affective nudges based on information provision generally exert short-lasting effects that tend to fade shortly after exposure (e.g., Brandon, Ferraro, List, Metcalfe, Price, Rundhammer, 2017, Calzolari, Nardotto, 2017).

**Fig. 9:**
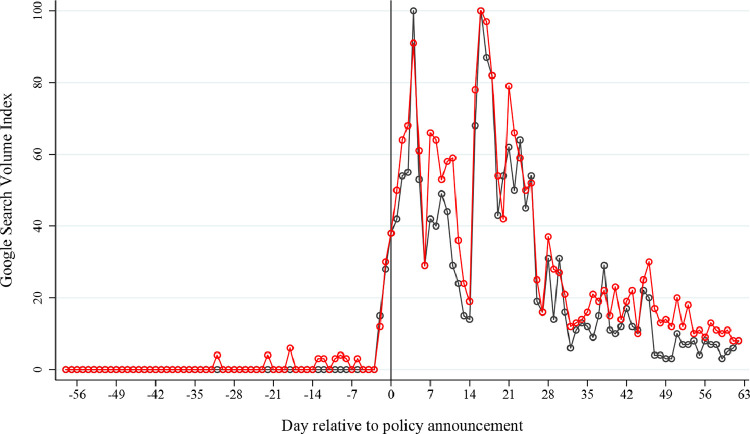
Online searches for food shopping vouchers. *Note:* The graph shows the search volume index (SVI) of searches for *COVID-19 food stamps* (black line) and *COVID-19 voucher* (red line). The SVI is a measure relative popularity on a 0–100 scale over the period February 1, - May 31, (2 months before and after the program’s announcement on March 30). The vertical line corresponds to the day of the announcement (0 on the x-axis). (For interpretation of the references to colour in this figure legend, the reader is referred to the web version of this article.)

The pattern of the treatment effects we document in Fig. 8 resembles the trend of the public’s interest in the aid program we detect through online searches. Figure 9 illustrates the daily volume of Google searches for ‘COVID-19 food stamps” (in red) and “COVID-19 voucher” (in black) from January 30 to May 30. We observe a spike in the week following the announcement. One week later, searches dropped with the Holy week and Easter holidays. In the third week, when authorities distributed most food stamps, online queries spiked again, suggesting that the food relief program was a particularly salient topic in the public’s interest. Searches then plateaued slightly below the spike for the following three weeks before definitively decreasing. Previous research has documented that Google searches for a specific topic substantially reflect the media coverage of that topic (see, for example, Jetter, 2017). The resemblance in the patterns of the treatment effects (Fig. 8) and the public’s interest in COVID-19 food stamps (Fig. 9) is striking and suggests that the media coverage may have helped the government levying the aid program to nudge compliance.

**Fig. A.2:**
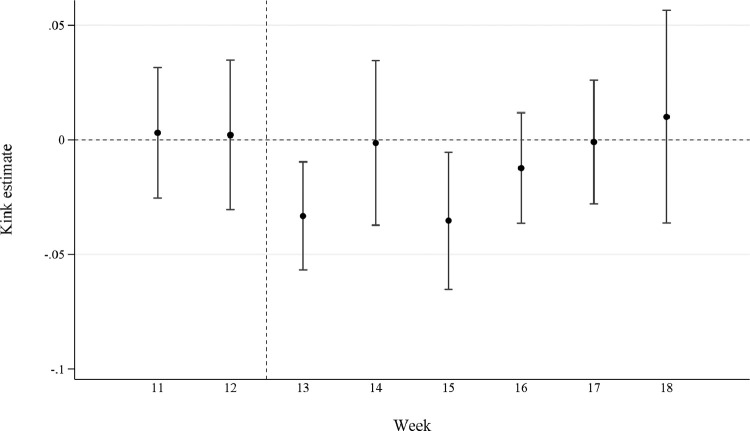
Program’s impact on mobility - pre and post policy announcement - quadratic polynomial. *Note:* The figure shows the kink estimates and the associated standard errors using the CCT optimal bandwidth and a quadratic polynomial. Results are displayed for the weeks preceding the policy announcement (11 and 12) and the weeks after (13 to 18). Week 11 refers to March 16–22, week 12 to March 23–29, week 13 to March 30-April 5, week 14 to April 6–12, week 15 to April 13–19, week 16 to April 20–26, week 17 to April 27-May 3, and week 18 to May 4–10.

Table 3 shows the point estimates of β for week 13 and 15, and includes alternative specifications without covariates (columns 1 and 3) and allowing for a second order polynomial for the term $f(R_{m})$ in Eq. (6) (columns 3 and 4). The full set of results for weeks 11–18 is presented in the Appendix in Table A.1. We also replicate the plot of coefficients in Fig. 8 using a second order polynomial. As we show in Fig. A.2 in the Appendix, the pattern of the results is virtually identical.

### 4.2 Robustness and placebo tests

**Fig. 10:**
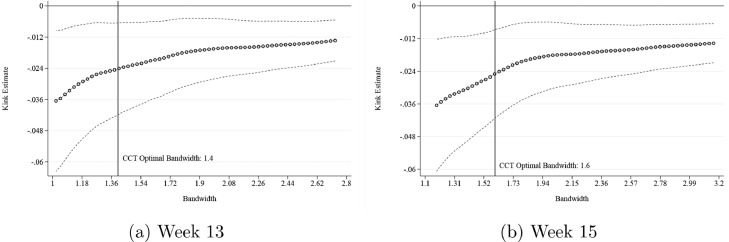
Kink estimates with varying bandwidths - linear polynomial. *Note:* The figure shows the point estimates and 90 percent confidence intervals for the main effect (on the y-axis), using a range of values for the bandwidth (on the x-axis), separately for week 13 and week 15. The figure shows that the absolute value of the point estimate is robustly around the one obtained using the CCT bandwidth. Narrowing the CCT bandwidth we obtain slightly larger negative values. The vertical lines represent CCT optimal bandwidths (Calonico et al., 2019a).

Essential robustness checks in the RKD regard the sensitivity of results to the bandwidth choice and the polynomial order. While the main results rely on the CCT optimal bandwidth, Fig. 10 illustrates how estimates change with varying bandwidths. We check the sensitivity of the results for weeks 13 and 15.

**Fig. A.3:**
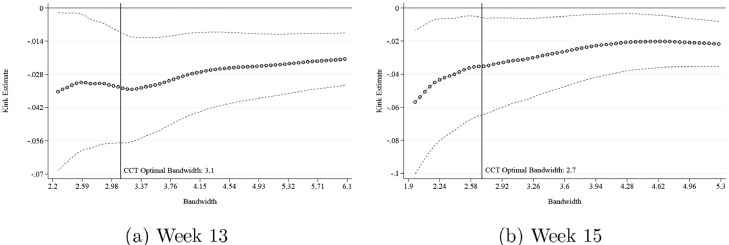
Kink estimates with varying bandwidths - quadratic polynomial. *Note:* The figure shows the point estimates and 90 percent confidence intervals for the main effect (on the y-axis), using a range of values for the bandwidth (on the x-axis), separately for week 13 and week 15. The figure shows that the absolute value of the point estimate is robustly around the one obtained using the CCT bandwidth. Narrowing the CCT bandwidth we obtain slightly larger negative values. The vertical lines represent CCT optimal bandwidths (Calonico et al., 2019a).

In Fig. 10, the vertical lines represent CCT optimal bandwidths (1.4 and 1.6). Overall, the estimates pattern corroborates our results, with stronger effects holding in the proximity of the cut-off and the coefficient stabilizing with distance from the threshold. In Fig. A.3, in the Appendix, we show similar patterns using a second-order polynomial. The effect is mostly stable along the spectrum of the considered bandwidths.

Although results using a quadratic polynomial are similar to our main estimates, we further test if nonlinearity in the relationship between mobility and our running variable is likely to induce spurious treatment effects. For this purpose, we follow Ganong and Jäger (2018) and compare the “true” kink estimates with a distribution of treatment effects obtained using “placebo cut-off points”. To implement the test, we consider 100 evenly spaced cut-off points in the region between −2.5 and 2.5, a broad range of values that includes the true cut-off point.

**Fig. 11:**
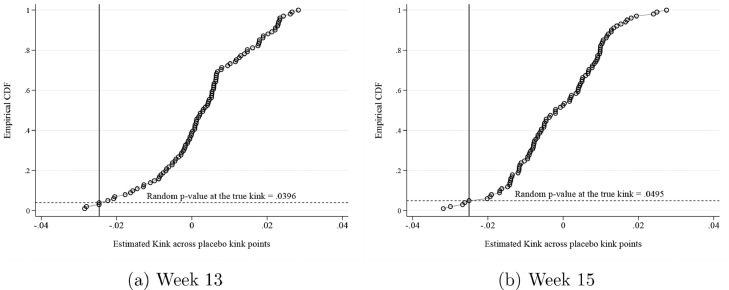
Permutation test Ganong and Jäger (2018) - linear polynomial. *Note:* The figures show the CDF of kink estimates using 100 evenly spaced placebo cut-off points in the interval [-2.5,2.5]. The vertical line corresponds to the value of the kink estimates at the true cut-off points. The horizontal dashed lines correspond to the values of the CDF at the true cut-off, i.e., the value of the permutation test.

**Fig. A.4:**
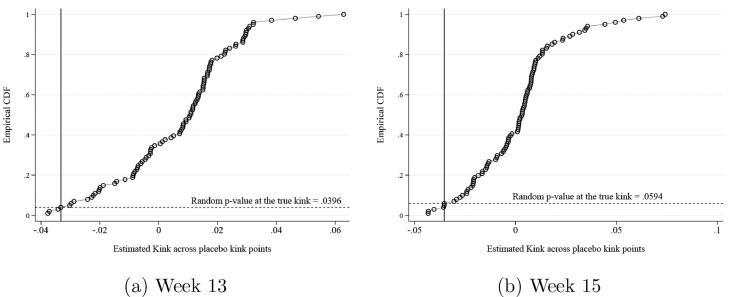
Permutation test Ganong and Jäger (2018) - quadratic polynomial. *Note:* The figures show the CDF of kink estimates using 100 evenly spaced placebo cut-off points in the interval [-2.5,2.5]. The vertical line corresponds to the value of the kink estimates at the true cut-off points. The horizontal dashed lines correspond to the values of the CDF at the true cut-off, i.e., the value of the permutation test.

After estimating separate kink regressions at each kink point using CCT optimal bandwidths, we compute the position of our main treatment effects in the conditional density function (CDF) of placebo estimates. Results of this procedure are shown in Fig. 11. The value of the CDF at the real cut-off corresponds to the value of the test statistics and is below 5% for both weeks. Figure A.4, in the Appendix, shows that similar results are obtained when a second order polynomial is used for the placebo estimates.

Finally, we conduct an exercise similar in spirit to the “Difference-in-Discontinuity” (Diff-in-Disc) approach proposed by Grembi et al. (2016). The policy context we address in our paper is inherently different from the standard Diff-in-Disc in two respects. On the one hand, the Diff-in-Disc approach is generally applied in a Regression Discontinuity setting, rather than a Regression Kink setting. More importantly, this empirical strategy is aimed at netting out pre-existing discontinuities to estimate the effect of a policy change at the cut-off point. In our setup there is no pre-existing policy with the same assignment mechanism, hence there is no reason to expect a change in slopes at the cut-off point before the treatment.^16^

**Fig. 12:**
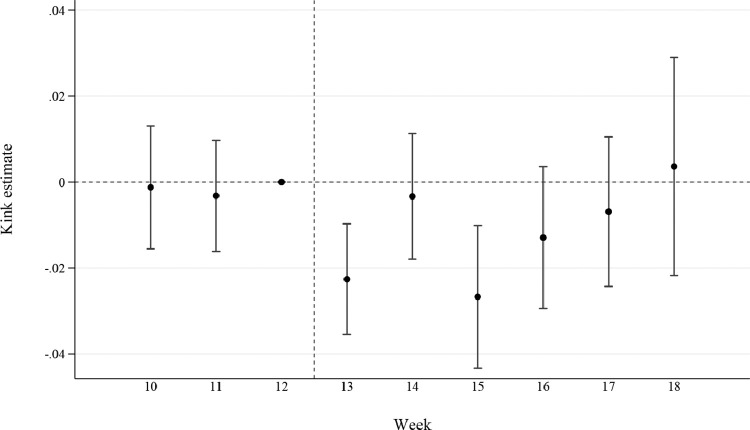
Program’s impact on mobility - linear polynomial and municipality Fixed Effects. *Note:* The figure shows the week-specific kink coefficients estimated jointly pooling all municipality-weeks observations (weeks 10–18). The polynomial order for each week is linear. The regression includes municipality fixed effect, week dummies, and the interaction terms between the covariates used in the main specification and week dummies. Week 12 is used as baseline.

Notwithstanding these differences, we adapt the “Diff-in-Disc” approach to the RKD setting to capture potential unobservable confounders that may affect our estimates. For this purpose, we jointly estimate our treatment effects in a pooled regression considering all weeks 10–18, i.e., the entire observation period. In this specification we exploit the availability of repeated observations for each municipality by including municipality fixed-effects and the interactions between the covariates used in the main specification and week indicators. Week 12 is used as baseline. In order to include just observations close to the cut-off point, the bandwidth used is the CCT optimal bandwidth for week 13.^17^ Results of this specification, shown if Fig. 12 are very similar to the ones obtained via separate (week-by-week) kink regressions.

### 4.3 Civic culture, fairness, and compliance

Municipalities entitled to the program’s *Quota B* received 11 percent additional resources for any given 1,000€ deviation from the threshold point. Our empirical analysis shows that these extra funds translated into an increase in compliance – i.e., an increase in the mobility differential relative to the baseline – of roughly 3 percentage points, so that the increase in transfers led to a 5 percent reduction in mobility.

Although the program was not intended to nudge compliance with shelter-in-place orders, improving the fairness of the COVID-19 policy response may have strengthened its overall legitimacy, helping citizens to internalize the societal benefits of restrictive measures. In this section, we delve deeper into the transmission mechanism of the effect and provide evidence suggesting that the fairness channel is empirically relevant.

Many authors connect the willingness to comply with rules with the belief that policy actions are appropriate and fair, as if a “social contract” binds citizens to obey their polity’s law (e.g., Tyler, 1990, Feld, Frey, 2007). Especially in emergencies, the legitimacy of measures imposing sacrifices for the common good crucially relies on the belief that authorities act in a fair and trustworthy manner (Tyler, 2006). The contractarian view implies that the public tends to reciprocate the government’s fairness, resulting in a higher compliance with rules (Frey et al., 2004).

However, citizens are not necessarily all alike in their approach to reciprocity and compliance. Besley (2020) shows that intrinsic reciprocity between the state and its citizens evolves based on the relative payoffs of civic-minded and materialist agents. As civic-minded individuals internalize social costs and benefits more, they get a higher payoff from the provision of public goods, resulting in a stronger incentive to reciprocate the state’s benevolence by complying with rules. This process tends to be self-reinforcing, as “A society with a strong civic culture encourages provision of public goods which increases the payoff of civic-minded citizens relative to materialists” (Besley, 2020, 1328). In other words, civic capital and fair and effective institutions support each other, as fair policies stimulate reciprocity and compliance among civic individuals, in turn strengthening the effectiveness of institutions. In such a virtuous cycle, civic-minded individuals can gain an evolutionary advantage over materialists.

Following this line of reasoning, we expect civic culture to catalyze the impact of relief policies, with more civic municipalities manifesting a stronger reaction to transfers and, therefore, a higher compliance. We test for this mechanism by exploiting several sources of municipality-level information to build a battery of civic capital indicators.

**Fig. A.5:**
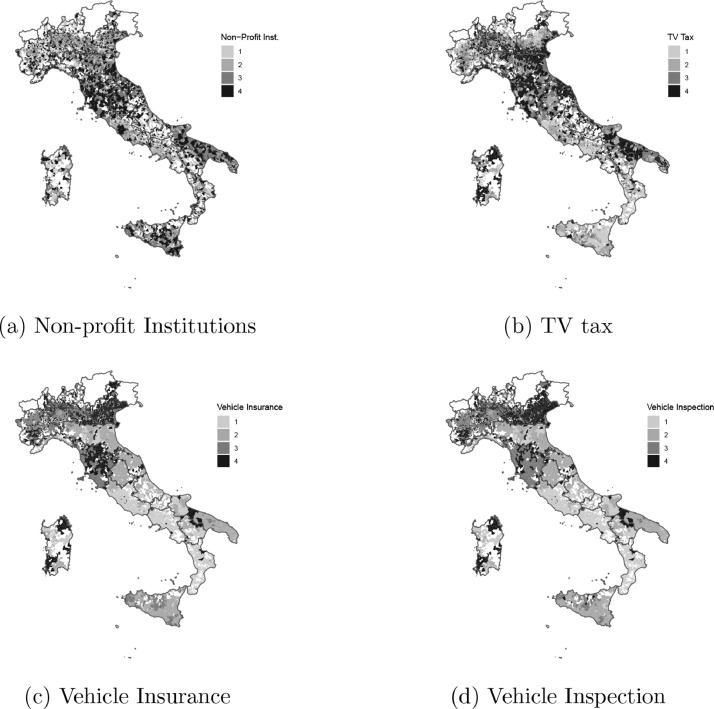
Geographical distribution of civic capital indicators. *Note:* The figure shows the geographical distribution of the civic capital indicators used in the heterogeneity analysis. Colors correspond to the four quartiles of the national distribution of each variable including the municipalities used for estimation.

In line with the abundant literature on civic capital, we first use census data to construct a municipality-level indicator of the density of voluntary associations (e.g., Knack, Keefer, 1997, Guiso, Sapienza, Zingales, 2016, Geraci, Nardotto, Reggiani, Sabatini, 2022). In their seminal work, Putnam et al. (1993) credit voluntary associations with the ability to promote cooperation, reciprocity, and public-spiritedness in their members. Then, we rely on administrative data to build three additional outcome-based measures of compliance behaviors that are specifically linked to civic mindedness, i.e., the share of vehicles covered by civil liability insurance, the share of vehicles that underwent the periodic motor vehicle inspection required by the Italian Ministry for Sustainable Infrastructure and Mobility, and the share of people regularly paying the Italian television tax. Although mandatory, these taxes are only occasionally enforced, making compliance strongly reliant on taxpayers’ civic-mindedness and good will, resulting in a remarkable variation across municipalities (Mariella, 2022, Durante, Mastrorocco, Minale, Snyder, 2022). Figure A.5 in the Appendix shows the geographical distribution of the four indicators of civic capital we use in the analysis.

Table 4 reports the results of a series of kink regressions. For each indicator, we split the sample into low and high civic capital municipalities (panels A and B, respectively) depending on their position relative to the median. In municipalities with low civic capital, the effect of the additional transfers is never statistically significant. In municipalities with a stronger civic culture, the relief program causes a significant increase in compliance. Treatment effects follow the same pattern observed in our main specification, with a significant reduction in mobility during the first week following the announcement, the effect fading out during the Holy Week, and a new significant reduction in the third week. The reduction in mobility stabilizes in the fourth week before becoming not statistically different from the one observed in municipalities below the cut-off in the following weeks. These results must be understood in relation to recent findings of an association between social capital and compliance with COVID-19 restrictive measures (Barrios, Benmelech, Hochberg, Sapienza, Zingales, 2021, Durante, Guiso, Gulino, 2021). We do not find a significant relationship between civic capital and mobility in a neighborhood of the specific threshold point that informed the distribution of funds in our policy case. However, the heterogeneity of the treatment effects allows us to catch another fragment of a complex picture. In areas poor of civic capital, citizens may be indifferent to relief measures improving the fairness of the crisis policy response. Otherwise, civic culture may work as a catalyst activating a behavioral response to the fairness of authorities, thereby encouraging compliance with emergency rules. Overall, the heterogeneity of the effects related to the strength of local civic culture suggests that the propensity for fairness and reciprocity played an empirically relevant role in triggering the compliance response of the public. This result is consistent with the implications of the contractarian view (Besley, 2020).

**Table 4:** Heterogeneity of the effects.

	Non-profit		TV		Vehicle		Vehicle	
	Institutions		Tax		Insurance		Inspection	
	Low	High	Low	High	Low	High	Low	High
$\beta_{1}^{13}$	0.0074	-0.0508***	-0.0087	-0.0315**	-0.0064	-0.0451**	0.0056	-0.0549**
	(0.0122)	(0.0160)	(0.0260)	(0.0134)	(0.0372)	(0.0222)	(0.0412)	(0.0240)
$\beta_{1}^{14}$	0.0159	-0.0190	0.0033	-0.0061	0.0145	-0.0208	0.0085	-0.0206
	(0.0125)	(0.0123)	(0.0213)	(0.0091)	(0.0365)	(0.0179)	(0.0425)	(0.0194)
$\beta_{1}^{15}$	-0.0055	-0.0434***	-0.0047	-0.0449***	0.0088	-0.0688**	0.0268	-0.0715***
	(0.0151)	(0.0133)	(0.0152)	(0.0156)	(0.0300)	(0.0271)	(0.0378)	(0.0275)
$\beta_{1}^{16}$	0.0110	-0.0291**	0.0074	-0.0246*	0.0119	-0.0344	0.0460	-0.0406*
	(0.0170)	(0.0124)	(0.0211)	(0.0133)	(0.0275)	(0.0223)	(0.0380)	(0.0224)
$\beta_{1}^{17}$	0.0249	-0.0317**	0.0014	-0.0096	-0.0070	-0.0294	0.0001	-0.0284
	(0.0224)	(0.0137)	(0.0243)	(0.0099)	(0.0304)	(0.0187)	(0.0391)	(0.0182)
$\beta_{1}^{18}$	0.0171	-0.0145	-0.0030	0.0004	-0.0057	-0.0092	-0.0001	-0.0145
	(0.0279)	(0.0240)	(0.0373)	(0.0119)	(0.0297)	(0.0201)	(0.0342)	(0.0209)

*Note:* The table shows coefficients and standard errors of separate kink regressions. For each variable considered, the sample is split in “High” and “Low” using the median of the variable distribution in the full sample. For each kink regression the bandwidth is computed according to CCT. Robust standard errors in parentheses. *p<.10, **p<.05, ***p<.01.

## 5 Conclusion

Emergency rules enacted to tackle extraordinary threats to public welfare can cause severe welfare losses for specific strata of the population, thereby undermining the overall fairness of the crisis policy response.

Since the beginning of the coronavirus crisis, confinement orders and mobility restrictions cause tremendous economic losses, worsen poverty and inequality, and threaten the social contract between citizens and the state. The economic hardship and the suppression of civil liberties can be unbearably unfair to impoverished individuals who cannot work from home and need to carry on income-generating activities.

In this paper, we exploited a sharp kink design in the allocation of aid funds across Italian municipalities to study the impact of relief policies on compliance with confinement measures. We provided robust evidence that, after the introduction of the program, compliance increased with the resources allocated to each municipality. The effect is economically sizeable and resists typical and more recently developed RKD robustness checks, such as bandwidth changes, with stronger effects holding in the proximity of the cut-off and the coefficient stabilizing with distance from the threshold.

Overall, the evidence on the relationship between poverty and social distancing in the coronavirus crisis points out the importance of tuning relief programs also in light of their potential impact on compliance. Programs targeted at individuals with lower incentives to comply may play a significant role in ensuring the observance of emergency rules. On a more general note, our evidence is consistent with the public economics literature that investigates the moral and social dynamics of tax compliance, suggesting that the perception of the political process as fair results in a stronger willingness to contribute to the welfare of the community (Feld, Frey, 2007, Besley, 2020).

Our results put forward actionable insights for policymakers. The finding that compliance was lower in poorer municipalities suggests that low-income agents face the most challenging difficulties in coping with stay-at-home orders. However, improving the fairness of the crisis policy response and alleviating the essential needs of economically disadvantaged groups can nudge social distancing substantially. This result suggests that relief programs must also be designed in light of their potential impact on compliance with emergency measures. Targeting relief measures at economically disadvantaged groups could more effectively support compliance than indiscriminate fiscal stimuli. More in general, compensation programs can improve the perceived fairness of the crisis policy response, thereby nudging people to internalize restrictive measures’ objectives and making them more willing to voluntarily bear sacrifices for the common good.

## Declaration of Competing Interest

The authors declare no conflict of interest.

## Figures and Tables

**Table A.1:** Kink estimates - all weeks.

			Mobility Index		
	Linear models			Quadratic models	
	(1)	(2)		(3)	(4)
**Panel A**			**Pre policy period**		
$\beta_{1}^{11}$	-0.0142	-0.0037		-0.0142	0.0030
	(0.0113)	(0.0091)		(0.0279)	(0.0174)
CCT Bandwidth	1.7975	1.457		2.3941	2.3891
N	2255	1881		2718	2659
Covariate	No	Yes		No	Yes
$\beta_{1}^{12}$	-0.0112	0.0021		-0.0087	0.0021
	(0.0141)	(0.0060)		(0.0309)	(0.0199)
CCT Bandwidth	1.535	2.1433		2.2119	2.2013
N	2000	2489		2600	2536
Covariate	No	Yes		No	Yes
**Panel B**			**Post policy period**		
$\beta_{1}^{13}$	-0.0344**	-0.0246**		-0.0466*	-0.0332**
	(0.0170)	(0.0110)		(0.0258)	(0.0144)
CCT Bandwidth	1.2616	1.3787		2.4413	3.0649
N	1721	1792		2751	3048
Covariate	No	Yes		No	Yes
$\beta_{1}^{14}$	-0.0163	-0.0018		-0.0240	-0.0014
	(0.0152)	(0.0095)		(0.0235)	(0.0219)
CCT Bandwidth	1.4316	1.6085		2.7267	2.2407
N	1894	2022		2945	2564
Covariate	No	Yes		No	Yes
$\beta_{1}^{15}$	-0.0400**	-0.0250**		-0.0490*	-0.0353*
	(0.0159)	(0.0099)		(0.0259)	(0.0182)
CCT Bandwidth	1.3043	1.5968		2.3791	2.6659
N	1752	2009		2708	2849
Covariate	No	Yes		No	Yes
$\beta_{1}^{16}$	-0.0229*	-0.0132		-0.0207	-0.0124
	(0.0130)	(0.0106)		(0.0246)	(0.0147)
CCT Bandwidth	1.5629	1.4997		2.5414	3.1494
N	2029	1927		2830	3092
Covariate	No	Yes		No	Yes
$\beta_{1}^{17}$	-0.0186	-0.0070		-0.0191	-0.0009
	(0.0138)	(0.0113)		(0.0243)	(0.0164)
CCT Bandwidth	1.4349	1.4358		2.4792	2.894
N	1897	1862		2782	2966
Covariate	No	Yes		No	Yes
$\beta_{1}^{18}$	-0.0051	-0.0006		0.0020	0.0100
	(0.0177)	(0.0176)		(0.0296)	(0.0283)
CCT Bandwidth	1.4917	1.3508		2.672	2.5151
N	1955	1768		2915	2750
Covariate	No	Yes		No	Yes

*Note:* The table contains coefficients and standard errors showing the estimated effect in percentage points variation in weekly mobility at the vicinity of the threshold point. The coefficients $\beta_{11}$ and $\beta_{12}$ refer to the weeks prior to the policy announcement. The 11th and 12th weeks cover the periods March 16–22 and March 23–29, respectively. Post-policy coefficients are $\beta_{14},\beta_{16},\beta_{17}$ and $\beta_{18}$ covering the periods April 6–12 (Holy week), April 13–19, April 20–26, April 27-May 3, May 4–10, respectively. The full set of controls includes population, municipality size, a dummy for urban areas, firm density, the per capita share of non-essential workers, the density of nonprofit organizations and the change in mobility during the first week of the lockdown. The bandwidths are selected using the procedure by Calonico et al. (2019a). Robust standard errors in parentheses. *p<.10, **p<.05, ***p<.01.

**Table A.2:** Criteria to allocate the food vouchers - Milan.

List	Criteria	Value	Points
1	Number of members aged < 18	0	0
		1	0.5
		2	1
		>2	1.5
2	Number of members aged > 64	0	0
		1	1
		>2	2
3	Number of members with disability	0	0
		1	0.5
		>2	1
4	Working condition	Employee	0
		Employee on layoff	0.5
		Self-employed	1.5
5	Yearly family income	€ 0–19.999	1
		€ 20,000-40,000	0.5
6	House owner	No	1
		Yes	0
7	Minimum income guaranteed by the state	Yes	0
		No	1
8	Other benefit	Yes	0
		No	1

## Footnotes

1 Authorities allowed police officers a discretionary power to assess the residents’ statements and fine transgressors € 200 (≃228$). On March 21, the government tightened the lockdown by shutting down all non-necessary businesses and industries. Given that authorities had caught more than 100,000 people outside for no good reason or lying on their forms, on March 25, the Italian Prime Minister announced an increase in lockdown fines up to € 3000 (≃3400$).

2 The Municipal Solidarity Fund was established in 2012 and supported through local taxes and it aimed at helping municipalities under public finance distress.

3 All the other relief programs allocated funds uniformly across targeted groups. For a summary, see, for example, https://www.bruegel.org/publications/datasets/covid-national-dataset/.

4 The decree of the Civil Protection Department’s head establishing the allocation of funds is available at the URL: https://www.trovanorme.salute.gov.it/norme/dettaglioAtto?id=73781.

5 See the decree https://www.gazzettaufficiale.it/eli/id/2020/03/30/20A01942/sg.

6 The vouchers could serve the purchase of essential goods such as products for personal hygiene, early childhood, and medical drugs. They did not entitle to the purchase of alcoholics and other non essential products. If the expenses were greater than the voucher value, the buyer had to pay for the difference. Vouchers could only be used by the holder or a person designated by them.

7 See details in the public call at https://www.comune.roma.it/web-resources/cms/documents/AVVISO_CONTIBUTO_ECONOMICO_COVID_rev5.pdf

8 For example, the municipality of Milan could distribute food stamps to only 13,475 households out of the 15,370 eligible ones due to funds constraints: https://www.comune.milano.it/-/coronavirus.-buoni-spesa-pubblicata-sul-sito-la-graduatoria-dei-beneficiari.

9 Aggregate municipal income tax data are available from the Department of Finance at https://www1.finanze.gov.it/finanze3/pagina_dichiarazioni/dichiarazioni.php.

10 Note that $pop_{N}$ is different from the total population in Italy ($pop_{IT}$) because municipalities in regions with special constitution (Aosta Valley, Trentino Alto Adige and Friuli Venezia Giulia) received different support regime.

11 Notice that the x-axis in Fig. 2 is expressed in € -thousands, with the value of the cut-off is equal to -1,466.

12 The daily mobility index at our disposal for each municipality can be expressed as:

$$MI_{m,d}=\left(mobility_{m,d}-mobility_{m,13jan-16Feb}\right)/mobility_{m,13jan-16Feb}$$

Importantly, we can only retrieve the LHS of the above equation, i.e., we do not have information on the underlying levels of mobility (RHS elements of the equation).

13 All municipalities in the autonomous regions of Aosta Valley, Trentino Alto Adige, and Friuli Venezia Giulia benefited from a different aid program with no kink schedule. See details on https://www.gazzettaufficiale.it/eli/id/2020/03/30/20A01942/sg.

14 The density of non-profit organizations is one of the oldest and more consolidated indicators of social capital. First proposed by Putnam et al. (1993), it was extensively adopted in the economics literature (e.g. Knack and Keefer, 1997 and Guiso et al., 2016). Putnam et al. (1993) credits the organizations we consider in the analysis with the ability to instill in their members habits of cooperation, solidarity, and public-spiritedness as opposed to the so-called Olson-type organizations that mostly have redistributive goals.

15 As explained in Section 3.1, the $\beta_{1}$ parameter measures, in the neighborhood of the cut-off, the effect of a one unit increase of the running variable on the outcome. Given the slope of the kink (0.586), $\beta_{1}$ also measures the effect of additional 0.586 per capital transfers induced by the one unit increase in the running variable.

16 Our estimates for pre-treatment weeks in Fig. 8 provide empirical support for the absence of a change in slopes in the pre-treatment period

17 We repeat this exercise using CCT optimal bandwidth computed on other weeks in the observation period, and one using the entire set of weeks. Results are virtually unchanged
